# Short-Range Electronic Interactions between Vanadium and Molybdenum in Bimetallic SAPO-5 Catalysts Revealed by Hyperfine Spectroscopy

**DOI:** 10.1021/acs.jpcc.3c01817

**Published:** 2023-05-31

**Authors:** Yu-Kai Liao, Valeria Lagostina, Enrico Salvadori, Martin Hartmann, Andreas Poeppl, Mario Chiesa

**Affiliations:** † Department of Chemistry and NIS Centre of Excellence, University of Turin, via Giuria 9, 10125 Torino, Italy; ‡ Felix Bloch Institute for Solid State Physics, Leipzig University, Linnéstr. 5, 04103 Leipzig, Germany; § Erlangen Center for Interface Research and Catalysis (ECRC), FAU Erlangen-Nürnberg, 91058 Erlangen, Germany

## Abstract

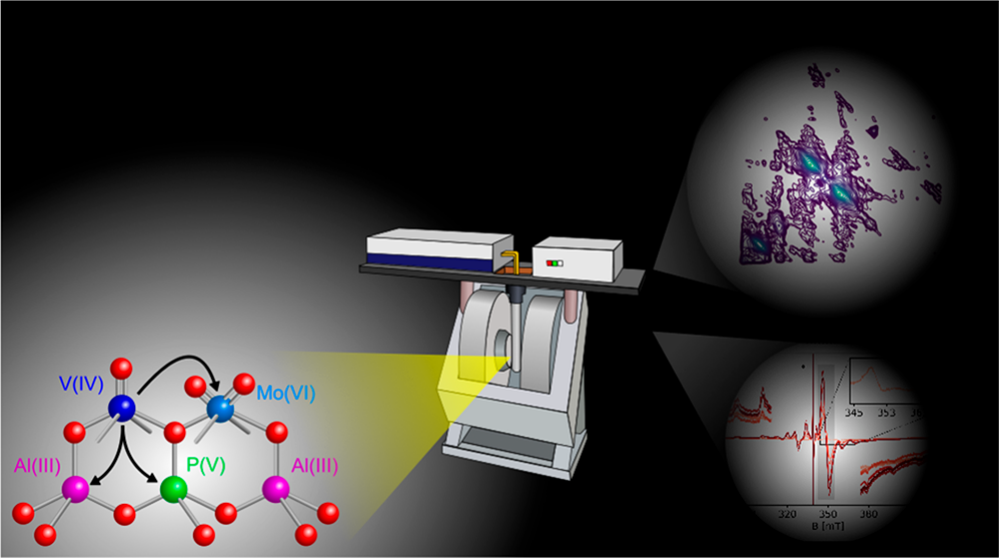

Engineering two cooperative sites into a catalyst implies the onset of synergistic effects related to the existence of short-range electronic interactions between two metal components. However, these interactions and the relative structure–property correlations are often difficult to obtain. Here we show that hyperfine spectroscopy has the potential to reveal the presence of V^4+^–O–Mo^6+^ linkages assessing the degree of spin density transfer from paramagnetic V^4+^ species to proximal oxo-bridged Mo^6+^ metal ions. The dimer species were prepared by adsorption of Mo(CO)_6_ in the pores of SAPO-5, followed by thermal decomposition and oxidation and subsequent grafting of anhydrous VCl_4_(g) followed by hydrolysis and dehydration. The metal species react with SAPO protons during the exchange process and generate new Lewis acid sites, which act as redox centers. X- and Q-band EPR and HYSCORE experiments have been employed to monitor the local environment of V^4+^ species obtaining direct evidence for spin delocalization over ^27^Al, ^31^P, ^95^Mo, and ^97^Mo nuclei, demonstrating the presence of bimetallic V–O–Mo well-defined structures.

## Introduction

Since the synthesis of titanium silicalite in 1983^1^ demonstrating that highly dispersed Ti species isolated at framework sites of microporous silica show unique properties for the selective oxidation of organic compounds, the isolation of transition-metal ions on high surface area supports has become a hot topic in catalysis. Metal-containing zeolites and zeotypes, in which metal centers are mainly isolated at crystallographic positions,^2,3^ indeed represent the most notable early example of what are now termed single metal catalysts (SACs).^4,5^

For example, tetrahedrally coordinated Ti(IV) centers in titanium silicate-1 (TS-1) and Ti-MCM-41 possess well-defined, single sites for activating a range of hydrocarbons and aromatics using hydrogen peroxide (H_2_O_2_) as oxidant.^6^ Analogous titanium sites can also be incorporated through isomorphous substitution of T-site atoms with Ti(IV) ions in the strictly alternating PO_4_^+^ and AlO_4_^–^ tetrahedra of aluminophosphate (AlPO) molecular sieves.^7^ Vanadium-doped AlPOs yet provide another compelling example of size- and shape-selective oxidation catalysts,^8^ while incorporation of silicon in so-called SAPOs introduces Brønsted functionalities for charge compensation.^9^ SAPO frameworks are built by alternating tetrahedral PO_4_ and AlO_4_ building units, for which some P^5+^ ions are isomorphously substituted by Si^4+^ ions at tetrahedral (T) sites. While AlPO frameworks are intrinsically neutral, the presence of isomorphously substituted silicon generates Si–OH–Al Brønsted acid sites similar to those present in aluminosilicate zeolites, which catalyze numerous reactions and can function as anchoring sites for metal species. The catalytic activity of SAPO can be further enhanced by engineering a second, redox-active site via simultaneous incorporation of two different transition-metal ions,^10,11^ providing these materials with a peculiar bifunctional character.^12^ These bicomponent composite catalysts are composed of one or more catalytically active sites and a functional support, in which the cooperation between transition-metal ions and the support materials can significantly enhance both the catalytic activity and selectivity of target products. Examples include polymerization catalysts^13,14^ as well as catalysts for partial oxidation processes where vanadium in tandem with other elements is a key component. Examples include V–Mo,^15,16^ V–Ti,^11,17^ and Co–Mo,^18^ all pointing to the presence of synergetic effects in the catalytic systems, which may play an important or decisive role in various catalytic oxidation reactions. However, little is known about the structures and properties of highly dispersed metals in SAPOs.^19^

The key for the remarkable reactivity of bimetallic dispersed species is the cooperative synergy between the two metal sites enabled by the optimal coordination structure enforced by the framework. This in turn is often a direct reflection of the synthetic protocols and specific reaction conditions. In fact, the metals can not only adopt different oxidation states but also in principle occupy both framework and extraframework positions. Distinguishing between the two situations is often not easy.

Clearly, subtle control of these bimetallic catalysts requires reliable characterization methods allowing to monitor in detail the local structural environment and the chemistry (catalytic potential) of the dopant ions. There exist a large number of spectroscopic techniques capable to give detailed insights into the coordination structure of oxo-bridged bimetallic species and successfully revealing their short-range structure such as X-ray spectroscopy^20^ or XPS.^21^ However, they either yield information averaged over the bulk or lack description of the intimate features of chemical bonding, which include covalency, ionicity, electron, and spin delocalization. For those particular cases involving metal oxidation states leading to “open-shell” electron configurations, electron paramagnetic resonance (EPR) spectroscopy and the associated hyperfine techniques of electron spin echo envelope modulation (ESEEM) and electron nuclear double resonance (ENDOR) can be particularly effective in characterizing TMIs in the AlPO’s structure and related materials as they allow to recover structural and bonding parameters at once.^22−25^

In this work, we study the coordination chemistry of V-SAPO and Mo/V-SAPO systems whereby the transition-metal ions have been grafted on the SAPO surface by gas phase reactions involving volatile metal precursors, namely VCl_4_ and Mo(CO)_6_, which are particularly well suited for detailed spectroscopic studies.^26,27^ In particular, we investigate the formed paramagnetic states (V^4+^ and Mo^5+^) using the electron spin localized on the metal as a probe to investigate the local environment of the metal species and their geometric and electronic structures.

## Materials and Methods

### Sample Preparations

The SAPO-5 was prepared by hydrothermal synthesis as reported elsewhere.^23,28^ To remove the template, the sample was calcined at 550 °C in air overnight. The AFI structure of the final product was verified by powder X-ray diffraction (Figure S1) obtained on a PANalytical Emryrean diffractometer employing Cu Kα radiation and is fully consistent with data from previous studies.^23,28^ After calcination, the sample was dehydrated by thermal treatment at 350 °C under dynamic vacuum (residual pressure <10^–4^ mbar) overnight in an EPR quartz cell.

Vanadium incorporation was obtained by an anhydrous vapor exchange process exposing the sample to the VCl_4_ vapors in a quartz cell equipped with an EPR tube. The cell was evacuated after the reaction to remove excess VCl_4_ and the reaction products (HCl), following established protocols.^29^

The bimetallic system was prepared by treating the calcined SAPO-5 powder with Mo(CO)_6_ (commercial Sigma-Aldrich) vapors at room temperature. The metal grafting was obtained by treating the sample under dynamic vacuum at 200 °C for 1 h. After this, molybdenum was fully oxidized (Mo^6+^) by increasing the temperatures from 100 to 300 °C in the presence of 100 mbar of molecular oxygen. Lastly, the system was contacted with VCl_4_ vapors as described above.

### EPR Spectroscopy

X-band (microwave frequency 9.5 GHz) continuous-wave (CW) EPR spectra were performed on a Bruker EMX spectrometer equipped with a cylindrical cavity. A modulation frequency of 100 kHz, a modulation amplitude of 0.2 mT, and a microwave power of 6.84 mW were used (15 dB). Q-band (microwave frequency 34 GHz) CW-EPR experiments were performed on a Bruker ELEXYS 580 EPR spectrometer, equipped with helium gas-flow CF935O cryostat from Oxford Inc. The magnetic field was measured with a Bruker ER035 M NMR gaussmeter. For Q-band measurements, the samples were introduced in the EPR tubes in a glovebox (O_2_ < 0.5 ppm, H_2_O < 0.5 ppm) and sealed in order to avoid contact with the atmosphere.

Q-band (microwave frequency 33.9 GHz) echo-detected field sweep (EDFS) spectra were recorded with the pulse sequence π/2−τ–π–τ–echo. Pulse lengths *t*_π__/2_ = 16 ns, *t*_π_ = 32 ns, a τ value of 200 ns, and a 1–2 kHz shot repetition rates were used.

Q-band (microwave frequency 33.9 GHz) standard hyperfine sublevel correlation (HYSCORE)^30^ or with remote detection (Remote-HYSCORE)^31^ experiments were performed with the pulse sequence π/2−τ–π/2–*t*_1_–π–*t*_2_–π/2−τ–echo (an additional sequence of storage/detection pulses π/2–*T*–π/2−τ_2_–π–τ_2_ was inserted before the echo for Remote-HYSCORE), applying a four-step phase cycle (eight-step for Remote-HYSCORE) for eliminating unwanted echoes. Microwave pulse lengths *t*_π__/2_ = 16 ns, *t*_π_ = 32 ns, and a shot repetition rate of 1–2 kHz were used. The *t*_1_ and *t*_2_ time intervals were incremented in steps of 8, 12, or 16 ns, starting from 100 ns giving a data matrix of 256 × 256, 170 × 170, or 128 × 128 points. For Remote-HYSCORE, τ_2_ = 200 ns was used to ensure the echo was out of the instrumental dead time for detection. The delay *T* between the standard HYSCORE sequence and the storage/detection sequence was selected the same order of magnitude as the phase memory time of the samples to ensure maximum sensitivity. The time traces of the HYSCORE spectra were baseline corrected with a third-order polynomial, apodized with a Hamming window, and zero filled. After two-dimensional Fourier transformation, the absolute value spectra were calculated. Spectra with different τ values were recorded, which are specified in the figure captions.

All EPR spectra were simulated employing the Easyspin package.^32^

## Results and Discussion

### Monometallic V/SAPO-5

**Figure 1:**
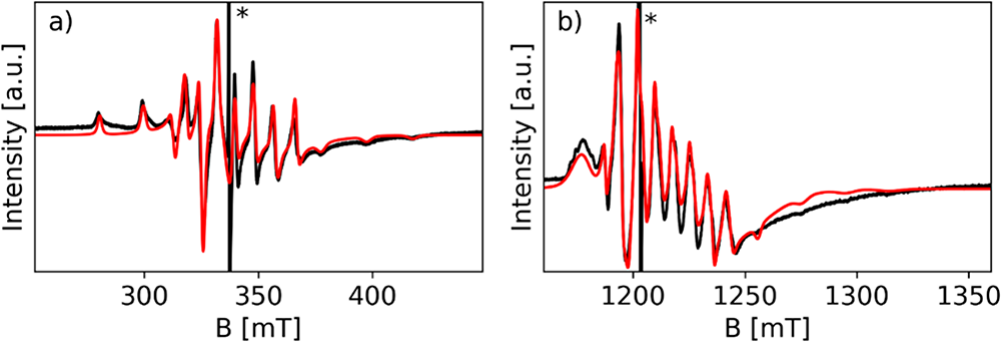
Experimental (black) and simulated (red) CW-EPR spectra of V/SAPO-5 recorded at (a) X-band and (b) Q-band. The spectra were recorded at room temperature (RT). The asterisks mark the signals of the residual coke radical in SAPO-5 after calcination.

After contacting the dehydrated SAPO-5 sample with VCl_4_ vapors, the characteristic 8-fold hyperfine splitting of all anisotropic components typical of V^4+^ (^51^V *I* = 7/2, abundance 99.76%) is observed in the EPR spectra recorded at X- and Q-band frequencies (Figure 1). The well-resolved ^51^V hyperfine pattern and the absence of broad absorption bands suggest a high dispersion of the paramagnetic species and absence of clustered or polymeric V^4+^ units. Spectral simulations were performed at both frequencies based on the following spin Hamiltonian:
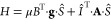
1The spectra recorded at the two frequencies could be satisfactorily simulated assuming a collinear axial model (i.e., *x* = *y* ≠ *z*, where *x*, *y*, and *z* refer to the principal directions of the **g** and **A** tensors using the spin-Hamiltonian parameters reported in Table 1.

**Table 1:** Spin-Hamiltonian Parameters Derived from the Simulation of the CW EPR Spectra^a^

	V^4+^						Mo^5+^				
	*g*_*x*_	*g*_*y*_	*g*_*z*_	|*A*_*x*_|	|*A*_*y*_|	|*A*_*z*_|	*g*_⊥_	*g*_∥_	|*A*_⊥_|	|*A*_∥_|	ref
VCl_4_–SAPO-5	1.9833 ± 0.001	1.9785 ± 0.001	1.9330 ± 0.001	213 ± 5	208 ± 5	533 ± 3					this work
VAPO-5	1.978	1.9734	1.9358	209	183	534					(22)
	1.978	1.9643	1.9375	207	178	515					
VCl_4_–ZSM-5	1.9843	1.9843	1.931	214	214	542					(29)
Mo/VCl_4_–SAPO-5	1.9840 ± 0.001	1.9785 ± 0.001	1.9335 ± 0.001	210 ± 5	195 ± 5	520 ± 5	1.9575 ± 0.002	1.9310 ± 0.003	100 ± 20	180 ± 30	this work
Mo– ZSM-5							1.945	1.878	190		(33)
Mo–SAPO-5							1.952	1.877			(34)

a Hyperfine coupling constant values are given in units of MHz. The spin Hamiltonian parameters were obtained by the simultaneous simulation of X- and Q-band spectra.

These values concur with typical values reported for VO^2+^ species,^22,29^ suggesting that the reaction of VCl_4_ with SAPO results in the formation of V^4+^–O^2–^ species, featuring a shorter V–O bond with typical characteristics of a vanadyl species. A similar situation has been reported in the case of reaction with ZSM-5 zeolites.^29^

Information on the local environment of the V^4+^ species can be elicited by measuring the hyperfine interaction with neighboring magnetically active nuclei. In the case of SAPO these are framework ^27^Al (*I* = 5/2) and ^31^P (*I* = 1/2) nuclei. The hyperfine couplings with these nuclei can be measured by means of HYSCORE, a two-dimensional experiment where correlations of nuclear transition frequencies in one electron spin (*m*_S_) manifold to nuclear transition frequencies in the other manifold are created by means of a strong mixing π pulse, providing an NMR spectrum of the coupled nuclei with sub-MHz resolution.

**Figure 2:**
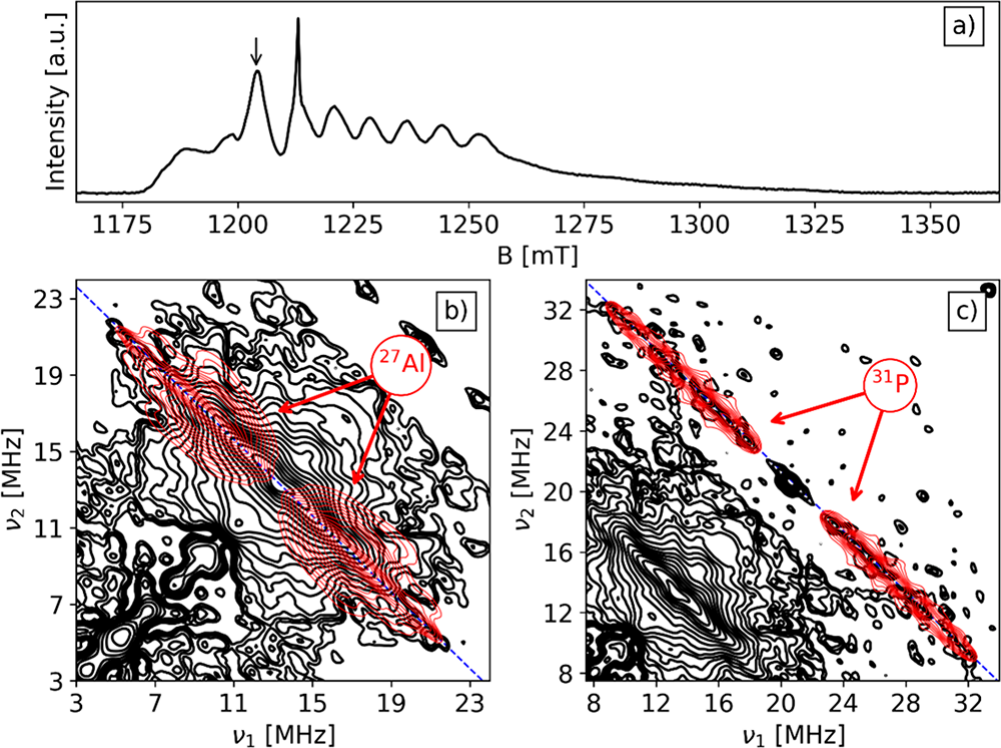
Q-band V/SAPO-5 spectra at 30 K of (a) EDFS spectrum and Remote-HYSCORE spectrum with τ = 24 ns at *B*_0_ = 1204 mT with simulation in red for (b) ^27^Al and (c) ^31^P. The HYSCORE contour levels in the two panels are adjusted according to the different signal levels of ^27^Al and ^31^P. The blue antidiagonal indicates the Larmor frequency of each nucleus.

The Q-band HYSCORE spectra of the V/SAPO-5 recorded at observer position *B*_0_ = 1204 mT are reported in Figure 2.

HYSCORE is a two-dimensional experiment which allows the recovery of the NMR transitions of magnetic nuclei interacting with the unpaired electron spin. In the experiment, correlation of nuclear frequencies in one electron spin (*m*_S_) manifold to nuclear frequencies in the other manifold is created by means of a mixing π pulse.^35^ The results are cross-peaks—or ridges in the powder spectrum—centered at the nuclear Larmor frequency and separated by the hyperfine interaction. The HYSCORE spectrum of the VO/SAPO-5 sample is characterized by two ridges centered symmetrically around ν_Al_ = 13.368 MHz and ν_P_ = 20.771 MHz coinciding with the ^27^Al and ^31^P Larmor frequencies. The width of the ridges corresponds to the maximum hyperfine coupling |2*T* + *a*_iso_| at a given observer position. Given the relatively low intensity, the HYSCORE spectra were symmetrized to better evaluate the ridge extension. Because the modulation depth of ^31^P and ^27^Al is significantly different, the signals corresponding to the two hyperfine interactions have been plotted separately in Figure 2b,c where the contour levels have been adjusted to optimum value for the two different nuclei. In Figure 2b, the ^27^Al ridge, with maximum extension of approximately 18 MHz, is plotted along with the corresponding simulation (red). Peaks appearing at lower frequencies in the symmetrized spectrum are instrumental artifacts. The simulation of the Al ridge shows the presence of two groups of Al species with isotropic hyperfine interaction parameter *a*_iso_ distributions of 3–9 and 13–15 MHz (see Table 2). The distribution of *a*_iso_ results from the structural fluctuation of the ligand environment around the paramagnetic metal center as previously reported.^13,22,23,36,37^ In our case, it can be associated with different extraframework sites at which the V^4+^ is grafted on the surface of SAPO-5. The two groups indicate that the vanadyl species interact with at least two different kind of Al nuclei. Spectra recorded at different magnetic field settings (Figure S2) show only little orientation dependence of the ^27^Al ridge signal, indicating that the hyperfine coupling is dominated by the isotropic Fermi contact term as already observed for similar systems.^22^ Considering the value of *a*_0_ = 3367.76 MHz for unit spin density on the ^27^Al 3s-orbital,^38^ the corresponding spin density in the Al 3s-orbital of the order of 0.17% and 0.42% can be estimated for the two set of nuclei, indicating the presence of V–O–Al linkages. Similar values were reported by some of us in the case of VO^2+^ extraframework species in VAPO-5 molecular sieves and Al_2_O_3_/TiCl_*x*_ catalyst.^13^

**Table 2:** Spin Hamiltonian Parameters Derived from the Simulation of the HYSCORE Experiments^a^

		^27^Al*				^31^P*					^95^ Mo				
		*a*_iso_ [MHz]	*T* [MHz]	α, β, γ [deg]		*a*_iso_ [MHz]	*T* [MHz]	ρ [MHz]	α, β, γ [deg]		*a*_iso_ [MHz]	*T* [MHz]	ρ [MHz]	α, β, γ [deg]	ref
Mo/VCl_4_–SAPO-5	^27^Al_1_	3–9	1–2		^31^P_1_	3–5	0.5–1	0		^95^Mo_1_	–3.0 ± 0.5	–1.3 ± 0.3	–0.5 ± 0.3	0, 90 ± 20, 0	this work
	^27^Al_2_	13–15	1–2		^31^P_2_	9–15	1–1.5	0							
					^31^P_3_	18–21	0.5–1	0							
VAPO-5		4.5	2.5	0, 90, 0		18	3.5	0.5	0, 80, 0						(22)
		2	1	0, 90, 0		6.7	2.15	0.5	80, 80, 0						
						2	0.8	0	0, 80, 0						
											^51^V				
VCl_4_–TiO_2_-101											6	0.5		0, 60, 0	(42)
VCl_4_–TiO_2_-001											7.17	3.46		0, 70, 0	

a ρ is an asymmetry parameter so that the traceless dipolar **T** tensor can be expressed as [−*T*+ρ; −*T*–ρ; +2*T*]. Because of the small anisotropy of the hyperfine interaction, Euler angles for ^27^Al and ^31^P are unresolved. *Identical parameters were obtained for VCl_4_/SAPO-5 samples.

The ^31^P HYSCORE signal (Figure 2c) is similar to that reported in the case of VAPO-5.^22^ The same analysis performed for the ^27^Al spectrum can be applied here, and considering the value of *a*_0_ = 10201.44 MHz, computed for unitary spin density in the P 3s-orbital, a spin density delocalization of the order of 0.04%, 0.11%, and 0.19% is estimated for three groups of P nuclei. The spin density transfers over the second shell coordinated Al and P are therefore of the same order, being primarily affected by the bond length and bonding angles of the V–O–L linkages where L can be either Al or P. The observation of relatively large coupling constants and similar spin density transfer over Al and P nuclei concur in indicating that the vanadyl species are anchored on the SAPO-5 surface at extra framework positions. This situation is clearly different from the case of framework incorporated vanadium species in VAPO-5 or TiAPO-5, where large hyperfine couplings were detected for P nuclei only, thus allowing for a clear discrimination between framework and extraframework transition-metal ions incorporation. Because of the doping of Si (10%) and the low natural abundance of ^29^Si (4.6%), no information can be retrieved on the proximity of Si atoms.

### Bimetallic Mo–V/SAPO-5

**Figure 3:**
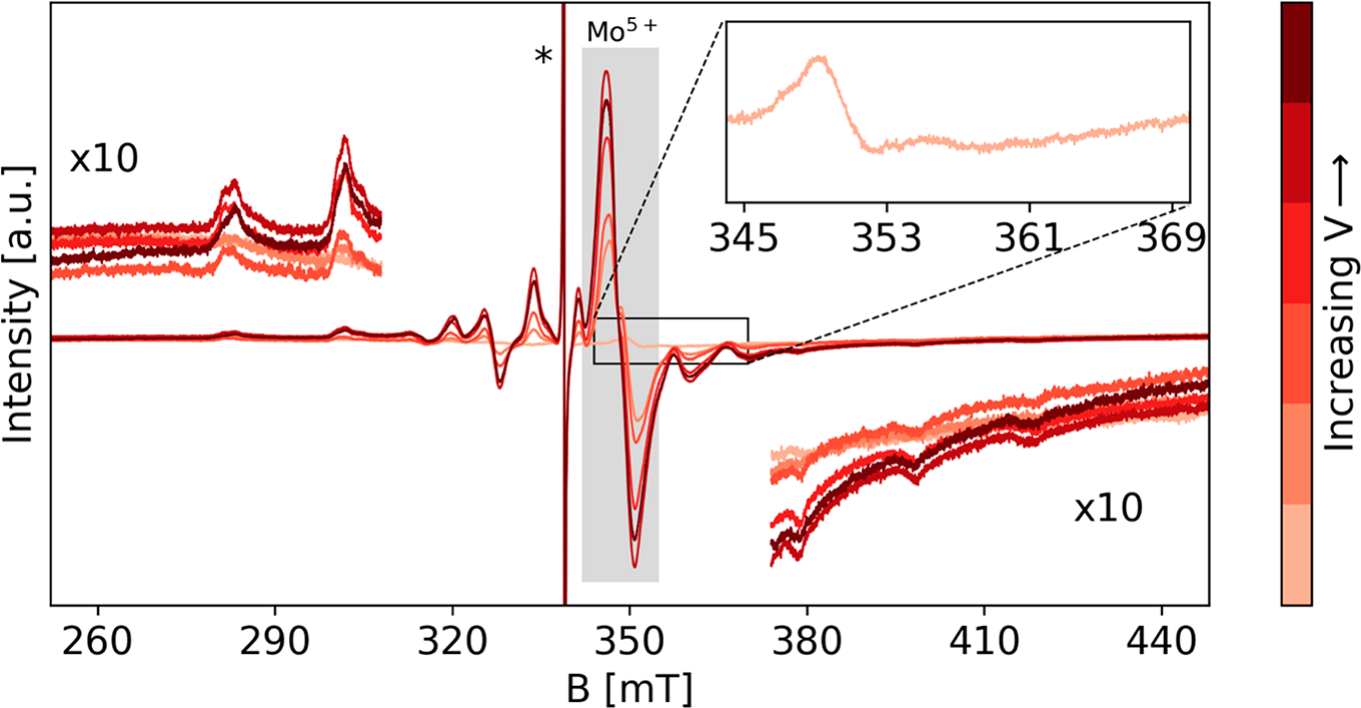
CW X-band EPR spectra of VCl_4_ deposited at increasing doses on Mo/SAPO-5. All the spectra are measured at room temperature. The inset shows the residual Mo^5+^ signal in the after the oxidation of the Mo, and the shaded area marks the Mo^5+^ signal that appears after the doses of VCl_4_. The asterisk marks the signal of the residual coke radical in SAPO-5 after calcination. Comparison of CW spectra of Mo/SAPO-5 and Mo–V/SAPO-5 after final VCl_4_ dosage is shown in Figure S3.

Similar experiments were performed on a SAPO-5 sample contacted with Mo(CO)_6_ followed by evacuation at 473 K and oxidation with O_2_ at 573 K. After oxidation, the EPR spectrum shows a weak signal in the spectral region between 345 and 365 mT (Figure 3). This can be attributed to residual Mo^5+^ characterized by an axial pattern with *g*_⊥_ = 1.940 and *g*_||_ = 1.876 typical of oxomolybdenum ions as (Mo(V)O_2_)^+^,^34,39,40^ while the dominant Mo species are in the EPR silent (VI) oxidation state (Mo(VI)O_2_)^2+^.

To investigate the possibility to establish short-range electronic interactions, which are at the basis of enhanced redox properties of bimetallic catalysts, VCl_4_ was evaporated on the Mo/SAPO-5 catalyst, with the objective of using paramagnetic V^4+^ as a spin probe.

**Figure 4:**
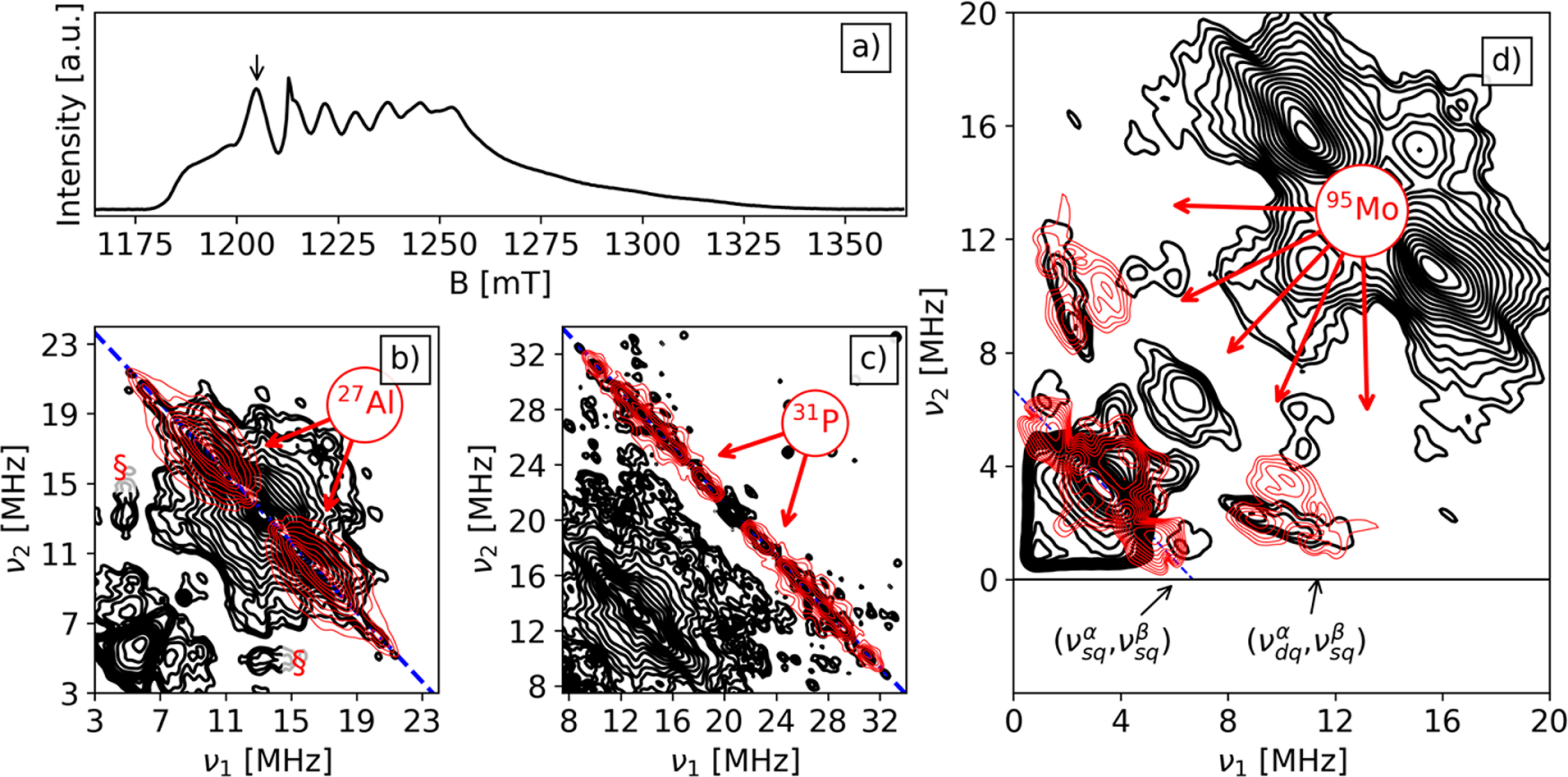
Q-band spectra at 30 K of (a) EDFS spectrum, b, c) Remote-HYSCORE spectrum (*B*_0_ = 1204 mT, τ = 24 ns) with nuclei simulations in red of (b) ^27^Al and (c) ^31^P, and (d) HYSCORE spectrum (*B*_0_ = 1204 mT, τ = 148 ns) with simulations in red of ^95^Mo. The HYSCORE contour levels are changed according to the different signal levels of ^27^Al and ^31^P. Larmor frequency of each nucleus in the (Remote-)HYSCORE spectra is marked in blue. The asterisk marks the signal of the residual coke radical in SAPO-5 after calcination. The symbol § in (b) indicates artifacts due to spectral symmetrization. Unsymmetrical spectra are shown in Figure S4.

In Figure 3 the X-band CW-EPR spectra are reported as a function of the VCl_4_ dose. As the amount of VCl_4_ increases together with the spectral features of V^4+^ illustrated in the V/SAPO-5 system (Figure 1), a clear increase of the Mo^5+^ EPR signal, evidenced by the gray shaded area in Figure 3, is observed. This fact clearly suggests a single electron transfer reaction from V^4+^ leading to the reduction of EPR silent Mo^6+^ species. Because the amount of V^4+^ is increased at each dose, we are not in the position to estimate the corresponding formation of the EPR silent V^5+^. The Mo^5+^ EPR signal is unstable over time, suggesting the complete oxidation of Mo as reported in the case of ZSM-5 zeolites.^41^ Due to the instability of the Mo^5+^ signal, advanced pulse EPR experiments could not be performed on this species. To ascertain the presence of V–O–Mo linkages, we performed Q-band HYSCORE experiments on the V^4+^ species in this Mo/SAPO-5 samples with deposited VCl_4_. The HYSCORE spectrum recorded at a magnetic field position indicated by the arrow on the EDFS spectrum is shown in Figure 4.

The HYSCORE spectrum displays a complex set of ridges and cross-peaks. Two sets (Figure 4b,c) are related to ^31^P and ^27^Al with couplings analogous to those discussed in the previous section and related to the interaction of V with nearby Al and P nuclei and will not be discussed further. Remarkably, at lower frequency a ridge with maximum extension of approximately 5.2 MHz appears on the diagonal at a frequency corresponding to the Mo Larmor frequency. Mo has two magnetically active isotopes both with *I* = 5/2—^95^Mo and ^97^Mo—relative abundances of 15.9% and 9.5%, respectively, and similar nuclear *g* factors, leading to Larmor frequencies of 3.36 and 3.43 MHz at the operational magnetic field (1204 mT). The ridge with maximum extension of about 5.2 MHz and centered at 3.4 MHz in the experimental spectrum is assigned to transitions involving the ±1/2 nuclear magnetic levels which are not affected (to first order) by the nuclear quadrupole coupling. In addition to this ridge, correlation peaks with |Δ*m*_*I*_| = 2 transitions are also observed appearing at frequencies 10.58 and 1.28 MHz. The presence of discrete cross-peaks and the extension of the |Δ*m*_*I*_| = 1 ridge indicate that the hyperfine coupling is not due to remote Mo nuclei but involves a non-negligible spin density transfer, i.e., the presence of an electronic interaction.^42^

**Figure 5:**
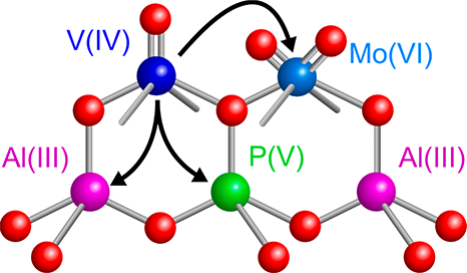
Schematic structure of the V/MO bimetallic site anchored at the surface of SAPO-5 with black arrows indicating the interaction of V with ^95,97^Mo, ^27^Al, and ^31^P.

Analysis of the ^31^P and ^27^Al HYSCORE spectra indicates that the spin density transfer over the two nuclei in the V–O–P and V–O–Al structures is of the same order. Based on this evidence and assuming the same situation holds for V–O–Mo linkages, the Mo hyperfine interaction was estimated assuming a spin density transfer of the order of 0.1% and using this value as a starting point for the simulation of the Mo transitions and trying to reach an agreement between the simulated and experimental spectra, while varying these parameters in fairly narrow limits.^43^ The nuclear quadrupole parameters were varied in broad limits in order to obtain an understanding of the role of this interaction in determining the HYSCORE cross-peaks. In doing this, we did not aspire to really fit the experimental HYSCORE spectrum but rather to achieve a qualitative resemblance between the simulations and experiments. The result is shown in Figure 4d where a reasonable simulation of the Mo spectral feature was obtained with a hyperfine tensor *A* = [−1.2, −2.2, −5.6] MHz (Table 2), where the sign of the tensor elements has been assumed negative, based on the negative nuclear *g* factor of both Mo isotopes. This results clearly proves that extraframework V and Mo species grafted on SAPO-5 via reaction of gas phase precursors led to isolated V and Mo centers characterized by different oxidation states. Under the experimental conditions, redox couples related to Mo^6+^/Mo^5+^ and V^5+^/V^4+^ species are present. Moreover, the detection of Mo hyperfine couplings corresponding to spin transfer of the order of 0.1% can be explained assuming that a fraction of the V^4+^ species interacts with oxidized covalently bound molybdenum ions (Mo^6+^), thus pointing to the presence of electronic interactions at short-range order compatible with V–O–Mo linkages. Similar bimetallic linkages have been reported recently by some of us at the surface of TiO_2_ involving mixed valence V^4+^–O–V^5+^ units, reminiscent of molecular V_2_O_3_^3+^ species.^42^ A schematic structure of the bimetallic site as derived from the EPR data is shown in Figure 5.

## Conclusions

Molecular precursors VCl_4_ and Mo(CO)_6_ have been used to disperse transition-metal ion species in SAPO-5 materials. EPR experiments demonstrate the formation of isolated and uniform V^4+^ -oxo species upon evaporation of VCl_4_. The formation of VO^2+^ likely proceeds by the surface reaction of chloride ligands in with Si–OH–Al, followed by hydrolysis of residual chloride, in a similar way as observed in aluminosilicate zeolites.^24,27^ HYSCORE experiments provide direct evidence for the chemical interaction of V^4+^ with both ^31^P and ^27^Al framework nuclei. From the measured hyperfine interactions, spin density transfers up to 0.2% and 0.4% for ^31^P and ^27^Al, respectively, are derived, consistent with V–O–P and V–O–Al linkages, indicative of the formation of extraframework metal species. In the case of the bimetallic V/Mo system, V^4+^ is exploited as a spin probe, revealing, in addition to hyperfine interaction couplings to ^31^P and ^27^Al, distinct cross-peaks in Q-band HYSCORE spectra, diagnostic of V^4+^–O–Mo^6+^ linkages. This is the most direct evidence that short-range electronic interactions between surface grafted metals are present, highlighting the potential of EPR and its related hyperfine techniques in the detailed characterization of these materials.

## Supporting Information Available

PXRD of the SAPO-5 and additional EPR spectra (PDF)
