# Supplementary material for: Short-Range Electronic Interactions between Vanadium and Molybdenum in Bimetallic SAPO-5 Catalysts Revealed by Hyperfine Spectroscopy
Source: J Phys Chem C Nanomater Interfaces. 2023 May 31;127(23):11103–10. doi: 10.1021/acs.jpcc.3c01817 (PMC10278125; doi:10.1021/acs.jpcc.3c01817)
Supplement: Supplementary file 1 — jp3c01817_si_001.pdf [file jp3c01817_si_001.pdf]

Short-range Electronic Interactions between Vanadium and Molybdenum in Bimetallic SAPO-5 Catalysts Revealed by Hyperfine Spectroscopy.

Yu-Kai Liao[1,2], Valeria Lagostina[1], Enrico Salvadori[1], Martin Hartmann[3], Andreas Poeppl[2], Mario Chiesa[1 *]

1 Department of Chemistry and NIS Centre of Excellence, University of Turin, via Giuria 9, 10125 Torino, Italy.

2 Felix Bloch Institute for Solid State Physics, Leipzig University, Linnéstr. 5, 04103 Leipzig, Germany

3 Erlangen Center for Interface Research and Catalysis (ECRC), FAU Erlangen-Nürnberg, 91058 Erlangen, Germany.

* Corresponding Author e-mail: mario.chiesa@unito.it

Supporting Information

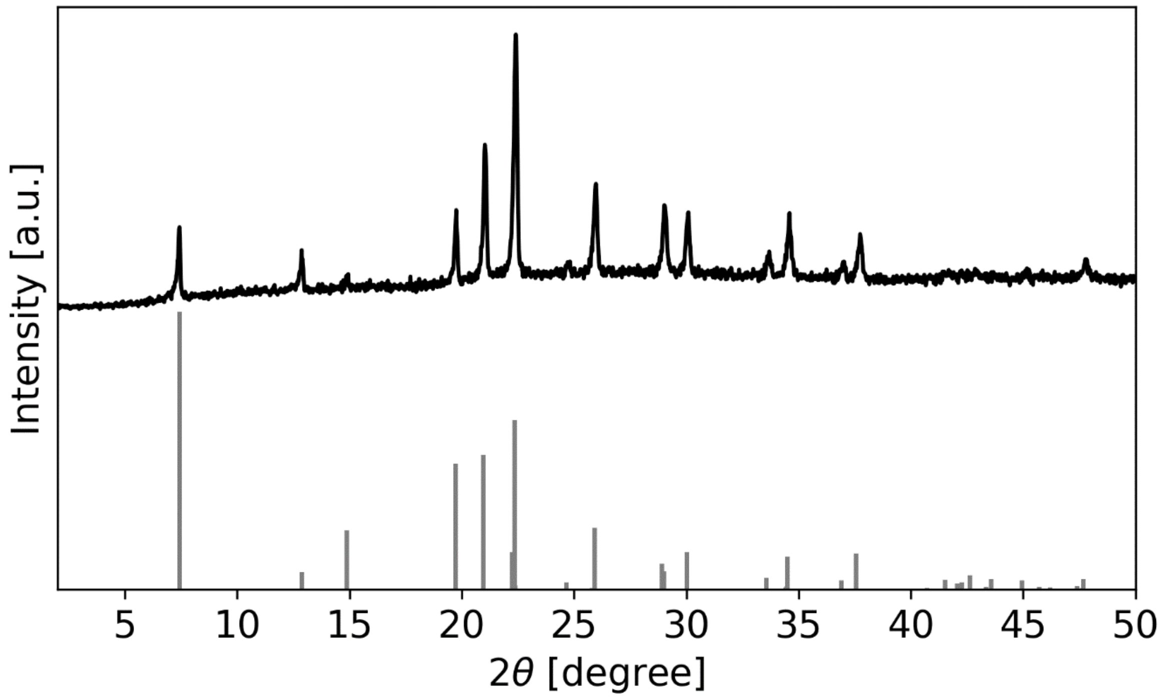

Figure S1 PXRD diffraction pattern of SAPO-5 measured after the calcination. Gray bars indicate the theoretical reflections of the AFI structure from the Database of Zeolite Structures. [1]

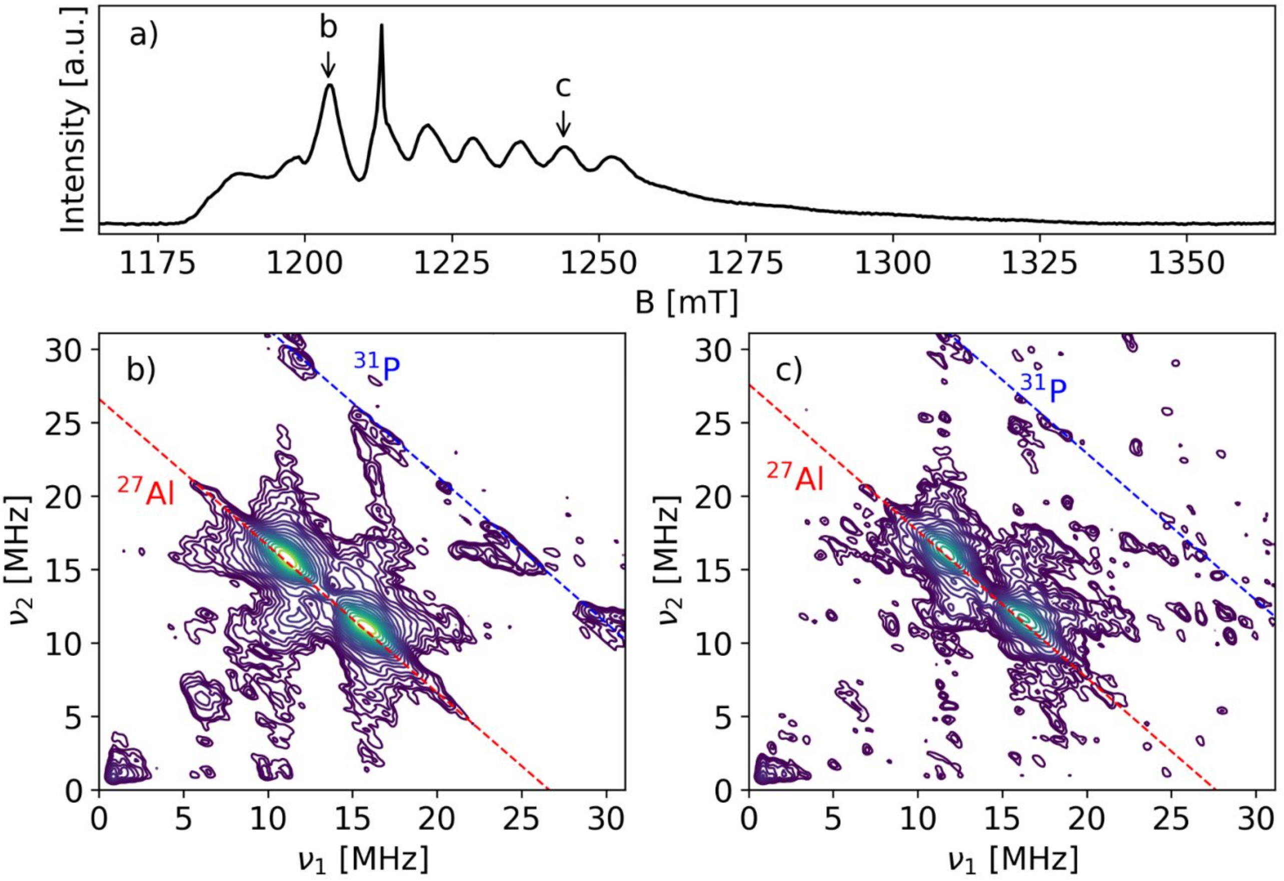

Figure S2 Q-band V/SAPO-5 spectra at 30 K of **a)** EDFS spectrum with arrows correspond to the fields where the HYSCORE spectra were measured. **b)** The HYSCORE spectra measured at 1204 mT superimposed with τ = 148, 218 ns and **c)** measured at 1244 mT superimposed with τ = 152, 224 ns. Blue and red dash lines indicate the Larmor frequencies of ^{31}P and ^{27}Al.

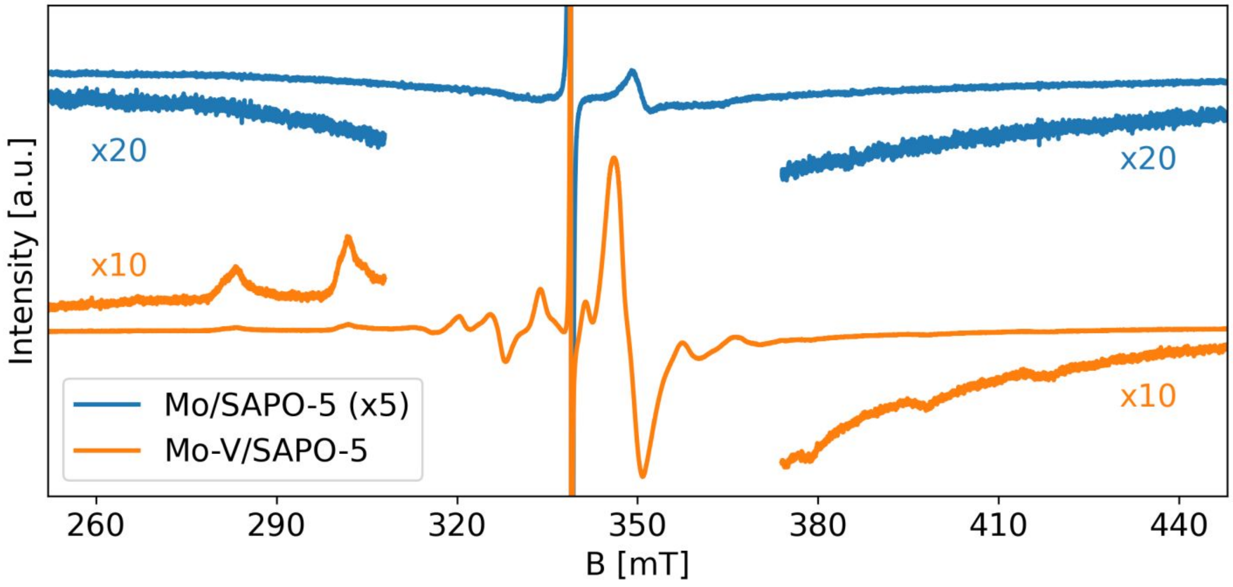

Figure S3 Comparison of the CW X-band EPR spectra of Mo/SAPO-5 and Mo-V/SAPO5 after the final VCl_4 dosage.

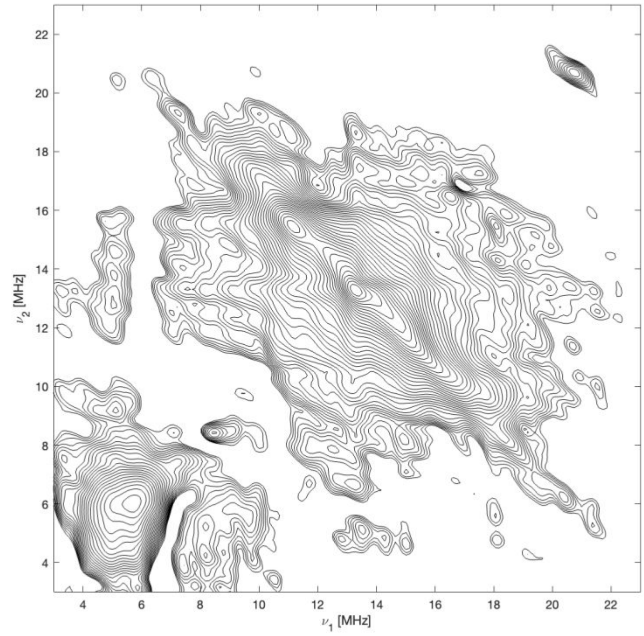

Figure S4 Unsymmetrized Q-band Remote-HYSCORE of Mo-V/SAPO-5 at 30 K (B_0=1204 mT, τ=24 ns).
